# Economy in the Poor-Law Infirmary

**Published:** 1907-04-20

**Authors:** 


### ECONOMY IN THE POOR-LAW INFIRMARY.

It is one of the curious features of Poor-law administration that whereas in the present day the need for economy is the theme of the reformer, the main danger in years gone by was an excessive and cruel parsimony. The regulations of the Local Government Board are a standing protest in favour of the rights of the poor with regard to food, clothing, baths, bedding, air space, warmth, and light. The poor man is entitled to his minimum, and even in institutions which have wisely abolished the allowance system, in favour of practically unlimited rations, the inmate has the legal right to insist on having his portion weighed out before him, should he believe himself to be defrauded of an ounce of bread. The inference is that there is more danger that the pauper shall come short than that he shall be too lavishly treated, for there appear to be no regulations extant prescribing that the inmate shall cost so much and no more.

There is a very real and instructive contrast between the economy of the voluntary hospital and that of the rate-supported institution. They are alike in this, that they both spend public money. But in the one case the money is contributed as a free gift by an altruistic section of the community from their surplus income, and in the other it is levied without consent from a population, often themselves in want of the good things which are bought in their name. On the face of it the rate-supported institution would seem likely to produce the better results from the point of view of economy. But does this prove to be the case in practice?

The hospital manager is undoubtedly tempted to lavishness in expenditure of a kind which readily appeals to the altruistic subscriber. Whatever is new in the way of invention or research has a strong attraction for those who are making personal sacrifices for the good of mankind. Whatever is on the side of efficiency, whatever makes for the sparing of suffering, or the building up of the weak, is in the eyes of the voluntary giver a matter for unsparing liberality. Accordingly the hospital manager vies with other institutions in installing costly plant, or in inaugurating new and expensive modes of treatment. Yet his resources are strictly limited, and he is hampered at every turn by the difficulty of meeting a mass of humdrum expenses about which the ordinary subscriber is indifferent to the verge of impatience. To a man who is anxious to spend a thousand pounds on installing a new electric lamp it would be irritating to be told that the cost of boarding only fifty nurses runs away with a similar sum every year, and that on the whole the hospital would be thankful to spend his money in this necessary direction rather than show the newest thing in lamps for treatment. No hospital manager would ever suggest such a course to an enthusiastic giver. But the manager is forced in consequence to reduce his budget for necessaries to the lowest point consistent with efficiency, and the wholly satisfactory result is apparent in the gradual reduction of domestic expenditure in hospitals year by year throughout the kingdom.

Now, the infirmary dare not advance too rapidly. Should the Guardians go beyond what is absolutely necessary for the bodily preservation of those under their care, they are confronted by a popular outcry on the part of those who are not only ignorant about the appliances and the skill necessary to restore the sick, but are resolutely indifferent. The main body of the ratepayers is essentially unaltruistic. Every step in the direction of more humane treatment of the sick—for the word "humane" in the present day cannot be realised without a good deal of expense—is won in the teeth of gigantic and stupid opposition, only justifiable when it is supported by a wide body of struggling workers barely able themselves to keep "off the rates." So far the tendency is to an economy which is in some directions of a deplorable character. But this is not all. There are many items in the budget of the infirmary which by the most parsimonious are admitted to be necessaries, and which, as we have shown, the Local Government Board, as the true guardian of the poor, takes care shall not be lacking. It is in these items that the Guardians are at a disadvantage compared with the hospital manager. Their supply of money is not limited to what is contributed in any particular year, and they are not compelled to look about for economies in one direction to make up for expenditure in another. They can for necessary articles, such as bacon and bread, boots and fuel, requisition what they please, and since the ratepayer has no means of scrutinising the accounts, and no inclination to do so even if he had a chance, there is no motive for economy.

It is not necessary to conclude that Guardians as a whole are in any degree reckless about the general expenditure of the institutions they manage. Apart from such cases of abuse as are never wanting under any system, however good, the Guardians of the poor to-day are probably more enlightened and more penetrated with a sense of public duty than has ever before been the case in the history of local government. But there is no restraining force in the direction of economy in small things such as, in the case of hospital managers, has effected in certain large hospitals savings of £20,000 during the last three years. And as it is the interest of no particular person to economise, there is laxity backed up by ignorance all along the line. One fact illustrates very clearly the strength and weakness of Poor-law economy. The Poor-law infirmary is usually worked with a staff of nurses which, even taking into full consideration the chronic character of most of the cases, is little short of dangerous both to the nurses and to the patients. The proportion of patients to each nurse is from ten to twelve compared with the three or four of the ordinary hospital. Yet in the infirmary the cost of boarding the officials often works out at almost double the sum found sufficient for the board of the hospital staff, and the savings which could be effected in this direction alone would suffice to increase the nursing staff by one-third, and yet leave something over.

### The Average Cost of Nursing Salaries per Bed.

"Matron" suggests that the variation observable in the matter of nursing salaries per bed is due to the practice in certain hospitals of receiving paying probationers. It is not, however, usual to deduct the sums paid by paying probationers from the total of salaries, nor would this conduce to clearer finance. But the question of paying the first year probationers or receiving them without salary is one which does materially affect the averages. The London Hospital, for instance, which stands highest in its average for salaries per bed, receives 170 first year probationers, exclusive of paying probationers, and gives them a salary of £12 each. The Radcliffe, Oxford, which has the lowest average among hospitals with medical schools, receives no nurses without a premium, and pays no salary till the second year. Yet it is clear that this is not the whole secret of the difference, for both Guy's and the Middlesex, which pay salaries to their first year probationers, show as low an average per bed as King's or the Royal Free, which do not commence to give a salary till the second year. Among the provincial hospitals the Birmingham General Hospital, paying no salary to first year probationers, has an average of £5 1s. per bed, while the Leeds General Infirmary, Sheffield Royal Infirmary, and the Bristol General Hospital, which pay salaries from the beginning, show respectively an average of £4 6s., £4 7s., and £5 8s. per bed. The Royal Infirmaries at Edinburgh, Dundee, and Aberdeen all pay salaries from the commencement of training, and their average rate of salaries per bed is £4 7s., £5, and £5 7s. The number of the staff seems to remain the strongest factor in determining the average cost of salaries.

